# Effects of News Frames on Perceived Risk, Emotions, and Learning

**DOI:** 10.1371/journal.pone.0079696

**Published:** 2013-11-04

**Authors:** Christine Otieno, Hans Spada, Alexander Renkl

**Affiliations:** 1 Department of Psychology: Educational and Developmental Psychology, University of Freiburg, Freiburg, Germany; 2 Department of Psychology: Cognition – Emotion - Communication, University of Freiburg, Freiburg, Germany; 3 Department of Psychology: Educational and Developmental Psychology, University of Freiburg, Freiburg, Germany; Cardiff University, United Kingdom

## Abstract

The media play a key role in forming opinions by influencing people´s understanding and perception of a topic. People gather information about topics of interest from the internet and print media, which employ various news frames to attract attention. One example of a common news frame is the human-interest frame, which emotionalizes and dramatizes information and often accentuates individual affectedness. Our study investigated effects of human-interest frames compared to a neutral-text condition with respect to perceived risk, emotions, and knowledge acquisition, and tested whether these effects can be "generalized" to common variants of the human-interest frame. Ninety-one participants read either one variant of the human-interest frame or a neutrally formulated version of a newspaper article describing the effects of invasive species in general and the Asian ladybug (an invasive species) in particular. The framing was achieved by varying the opening and concluding paragraphs (about invasive species), as well as the headline. The core text (about the Asian ladybug) was the same across all conditions. All outcome variables on framing effects referred to this common core text. We found that all versions of the human-interest frame increased perceived risk and the strength of negative emotions compared to the neutral text. Furthermore, participants in the human-interest frame condition displayed better (quantitative) learning outcomes but also biased knowledge, highlighting a potential dilemma: Human-interest frames may increase learning, but they also lead to a rather unbalanced view of the given topic on a “deeper level”.

## Introduction

The media play a key role in forming opinions by influencing people´s understanding and perception of a topic. A research field highly relevant to understanding media effects on information processing refers to news frames. Generally speaking, news frames present information in a way that emphasizes certain aspects of a topic, thus making those informational aspects more salient than others [1]. Research on framing effects found influences of news frames on opinion formation [2], recall [3,4], responsibility attribution [5], and the emotional reactions [6] of mass-media recipients. Moreover, educational psychology findings reveal that such emotional reactions may influence learning [7,8]. This study attempts to combine framing-research theories and findings with theories of cognitive and educational psychology to clarify framing effects on the perception of risk, emotions, and learning. More specifically, we aimed to test for (a) framing effects in human-interest frames compared to a neutral text independent of content, and (b), whether these effects can be "generalized" to different variants of the human-interest frame (i.e., variants with an emphasis on emotionalization, dramatization, or personalization). Finally, we explored (c) potential mediations of perception of risk on emotions and of emotions on learning outcomes. 

### Framing and Framing Effects

Framing is concerned with the presentation of information. Therefore, framing effects can be defined as the effects of differences in an issue’s presentation on how people perceive and understand a topic [5,9,10]. These effects are moderated by factors such as the recipients' issue involvement, perceived importance [11–13], and prior knowledge [13–16]. 

One classic example of framing research is the “Asian disease study” by Kahneman and Tversky [17]: Participants were much more likely to choose the variation of a program to fight an “Asian disease” naming a number of certain survivors over a factually equivalent program naming a number of certain deaths. This kind of framing is also referred to as *equivalence framing*, that is, framing which directs attention to different aspects in a factually identical description [18]. Equivalence framing should be distinguished from *emphasis framing* which suggests a certain perspective by emphasizing specific, equally true, yet different subsets of aspects of a topic without the assumption that the information presented is necessarily factually equivalent [19,20]. In other words, the main difference is that while equivalence frames present factually equivalent information from different perspectives, emphasis frames present information that suggests a certain perspective, yet is not necessarily factually equivalent between different frames. Emphasis framing is common in public media and is therefore often found in research on media effects and specifically *news frames*.

### Effects of news frames on perception of risk, emotions, and learning

News frames exert their effects by emphasizing the “salience of different aspects of a topic” [1], p. 53 (cf. emphasis frame). Lang and colleagues [21] highlighted the importance of psychological theories for understanding cognitive processes underlying media effects in general, and framing effects in particular. Specifically, theories of mental models [22] and situation models in text comprehension [23] provide important frameworks for understanding cognitive media effects [24]. A situation model is essentially a mental model of the situation or issues described in the text, which the reader builds by enriching the information given in the text (text-base) with prior knowledge. Additionally, the construction-integration model of text comprehension proposes that linguistic cues guide the readers´ attention while reading the text, thereby influencing the focus of the situation model [25]. Despite the importance of cognitive theories to explain framing effects, there are few studies directly addressing and systematically measuring cognitive effects of news frames on information processing and learning [26,27]. The study of Berinsky and Kinder [28] is one of the rare examples in which framing effects on learning were investigated and embedded in a cognitive theory. Their results are related to psychological theories on information processing [23,29] and corroborate the assumption of framing theories that an event’s specific framing helps people understand complex sequences of events and create a coherent story [18,30,31]. Emphasizing a particular storyline may also direct readers´ attention toward certain aspects of the information while drawing it away from others, leading to an effect similar to the *seductive details effect* in multimedia learning [32]. However, while the term *seductive details* is usually used for interesting yet irrelevant informational aspects, framing draws attention to relevant aspects of information while detracting it away from other equally relevant aspects. In relation to the seductive details effect, this effect may therefore be designated as a *seductive emphasis effect* [33]. 

Studies about framing effects in the media identified the following widely used journalistic news frames: the conflict frame, attribution of responsibility frame, morality frame, economic consequences frame, and human-interest frame [34–36]. Responsibility frames emphasize the responsibility of an individual or a group for a certain event or outcome, while the morality frame presents an issue in the context of religious or ethical prescriptions. The economic consequences frame focuses on economic consequences for individuals or groups. Finally, human-interest frames use specific features, such as dramatizing or emotionalizing vocabulary to catch the audience´s attention and often lend the story an individual, personalized touch [35]. 

Although human-interest frames are widely used in news stories, there is little research on the specific effects of this frame on emotions, perception of risk, and learning [36]. From an experimental perspective, the human-interest frame is particularly interesting as it consists of different features, which have been studied separately before [6,21,33,37,38]. However, we were interested, if the human-interest frame as a whole elicits similar effects as its separate features. Therefore, it seems promising to manipulate the strength of these features, emphasising one or the other more, while keeping all necessary characteristics of the human-interest frame as defined by Semetko and Valkenburg [35], and to test if effects can be “generalized” over different variants of the frame. This variation of emphasis allows for controlling that it is really “the sum of its parts” and not merely one of the features of the frame that produces typical human-interest frame effects on emotions, perception of risk, and information processing. 

Different journalistic news frames are known to exert differential emotional responses [6] and to alter perceived risk [33]. Appraisal theories of emotion and the model of social amplification of risk respectively, may explain framing effects on emotions and perception of risk. Within the framework of appraisal theories, appraisal is defined as the cognitive evaluation of a situation with respect to the demands, constraints, and resources in the situation on the one hand, and the individual’s goals and resources on the other [39–41], (for an overview see [42]:). Similarly, the cognitive evaluation of a situation, especially the perceived severity and probability of negative outcomes, influences emotions and perceived risk [43]. The cognitive evaluation (or appraisal) of the situation is likely to be influenced in both cases by the difference in the salience of certain aspects achieved by framing. Additionally, the model of social amplification of risk assumes a strong media influence on both the public discourse and each individual’s and the social group’s perceived risk [44]. Recent theories on the perception of risk additionally assume an interaction between the perception of risk and emotions [45,46]. For example, perceived risk may be considered a specific form of appraising a situation (as risky versus not so risky), and therefore potentially influence emotions [47]. 

Additionally, connecting emotions with cognitive theories, the personality systems interaction theory assumes that positive emotions such as happiness activate rather wide semantic fields and lead to holistic information processing, while negative emotions such as anger lead to a focus on rather narrow informational aspects, that is, the spread of activation is limited to close associates and dominant words [48]. Assuming that human-interest frames lead to stronger negative emotions, this could have two effects on learning: First, the higher activation level should lead to better (quantitative) learning outcome in general (cf. cognitive motivational mediation model of emotions [7]). Second, restricting the spread of activation should lead to focusing on only those aspects that seem to be particularly relevant to the frame. Both might be particularly true for complex and controversial topics that demand substantive processing [49]. Additionally, specifically moderate arousal (i.e., arousal that is neither specifically high or low) may enhance memory of frame-specific, negative information [38].

One problematic aspect in most studies investigating news frame effects is, that the systematically different information about the topics at hand - which results from the fact that the contents of emphasis frames naturally differ to some extent from one another - makes it difficult to distinguish between content and actual framing effects. For example, Nelson and colleagues [50] framed a Ku Klux Klan rally as either a free speech issue or a disruption of public order to investigate framing effects on, for example, tolerance of the Ku Klux Klan. Igartua and colleagues [15] framed the issue of an increase in immigration in Spain as either causing positive economic consequences or a rise in criminality, and measured framing effects on attitudes towards immigrants, emotions, and cognition. 

Some researchers aimed to separate content from framing effects by implementing the news frames only in the headline, introduction and concluding paragraphs of newspaper articles while keeping the core component of the text the same for all conditions [3,4]. Valkenburg and colleagues [4] compared effects of different news frames (conflict, human interest, responsibility, and economic consequences) and one control condition, which consisted only of the common core text. Additionally, they used two different cover stories, namely the introduction of the Euro and increasing crime rates. They found, in accordance with Price and colleagues [3], that readers' thoughts referring to the text as a whole (differently framed parts as well as common parts) are strongly influenced by the frame. Additionally, they found a negative effect on recall measured by multiple-choice test (only items referring to the information identical for all conditions) for the human-interest frame in comparison to all other frames, specifically for the crime story but not the introduction-of-the-Euro story. They hypothesized, that it was likely the emotional nature of the topic that led to stronger emotional reactions (i.e., high emotional arousal) and therefore poorer recall. 

More recently, Von Sikorski and Schierl [13] attempted to separate the confounding of frame and content by systematically varying only those sentences that contained frame-relevant information while keeping the core text constant. They compared reports on disability sports concerning their effects on the attitude towards an athlete described in the texts 1 frame placed emphasis on the athlete’s performance, while the other emphasized his dependency on public support. Additionally, they introduced a third, so called “mixed frame” providing information from both main frames. As expected, participants´ attitudes in the mixed-frame condition fell in between the attitudes reported in both main-frame conditions. This result indicates that what is sometimes presented as a “framing effect” in research employing emphasis frames might at least in part be a “content effect”. 

### The Present Study

Our study focuses on the effects of the human-interest frame compared to a neutral text on perceived risk, emotions, and learning. More specifically, we compared a neutral text (control condition) with three variants of the human-interest frame, which contained an emphasis on the different features constituting the human-interest frame, that is, either dramatizing or emotionalizing vocabulary, or an individualized storyline. We did not attempt to completely separate those three aspects of the human-interest frame but rather to present ecologically valid versions of the frame with specific emphases. Hence, we did not investigate specific differences among the three versions of the human-interest frame but rather used the variation to test for the "generalizability" of our results to common variants of the human-interest frame instead of comparing only one kind of human-interest frame to a neutral text. That is, the variations in the human-interest frames allowed us to control for the possibility, that merely one feature of the human-interest frame is responsible for human-interest frame effects. 

In order to measure framing effects independent of content, we followed the design of Valkenburg and colleagues [4] and kept the core component of our text constant for all conditions and only varied the headline, introduction, and concluding paragraph. To avoid influences of text length, also participants in our control condition were presented with an introduction and conclusion, which, however, was written in rather neutral language not employing any frame-specific features. Additionally, we separated the differently framed introductions and concluding paragraphs thematically. More specifically, we used the topic of invasive species in the differently framed introduction and conclusion sections and the topic of the Asian ladybug, one example for invasive species, in the common core text. Reported framing effects refer to information given in the invariable core text only, unless explicitly stated otherwise. The topics of invasive species in general and the Asian ladybug in particular seem particularly suited for investigating framing effects as they are highly complex topics about which the general public has little prior knowledge and the scientific community shares conflicting opinions [51,52]. Furthermore, we controlled for specific prior knowledge concerning invasive species and the Asian ladybug, as well as prior perceived risk concerning invasive species (no prior perceived risk measures were collected for the Asian ladybug, as the issue was new to most of our participants). Specifically, we tested the following hypotheses: 

First, we anticipated framing effects on perceived risk and emotions. More specifically, we assumed that perceived risk would be higher for all variants of the human-interest frame compared to the neutral-text condition (*perceived-risk hypothesis*). In addition, negative emotions would be stronger for all variants of the human-interest frame compared to the neutral-text condition (*emotions hypothesis*). Finally, we assumed that framing effects on emotions are mediated by perceived risk (*mediation-by-risk hypothesis*). 

Second, we expected framing effects on learning outcomes. More specifically, we assumed that participants in the three human-interest frame conditions would differ in the amount of knowledge they acquire compared to those in the neutral-text condition (*amount-of-learning hypothesis*). Additionally, participants in the three human-interest frame conditions would prioritize negative information and disregard positive information (*prioritization hypothesis*). Finally, we assumed that framing effects on learning and prioritization are mediated by the emotional state (*mediation-by-emotion hypotheses*). 

To answer the question whether effects are "generalizable" to different variants of the human-interest frame or whether it is specifically one feature that mainly exerts respective effects, we also tested for differences between the human-interest frame conditions with respect to the perceived risk and emotions hypotheses, and the amount-of-learning and prioritization hypotheses. However, we did not necessarily expect such differences.

## Materials and Methods

### Ethics statement

All participants volunteered and provided verbal informed consent. The study was conducted in accordance with the German Psychological Society (DGPs) ethical guidelines (2004, CIII) as well as APA ethical standards. According to the German Psychological Society´s ethical commission, approval from an institutional research board only needs to be obtained, if funding is subject to ethical approval by an Institutional Review Board. This research was reviewed and approved by the Ministry of Science, Research, and the Arts of Baden - Württemberg, Germany (grant number 33-720.281/34), which did not require additional Institutional Review Board approval. All data was collected and analyzed anonymously.

### Participants and design

Participants were recruited at the main campus of a German university. Ninety-one students of law (51.6%), medical science (22%), social studies (20.9%), and economics (5.5%; age: M = 20.88 years, SD = 2.97; 30.8% male and 69.2% female) participated in our study. All participants received €10 as compensation. The participants were randomly assigned to one of the four conditions (emotionalizing, dramatizing, or individualizing human-interest frame and neutral text) in a pretest-posttest design. 

### Materials

#### Stimulus material

Our stimulus material was designed to resemble a real-life newspaper article about the Asian ladybug as an example of an invasive species. Invasive species are animals and plants introduced to or occupying “new” regions. The Asian ladybug is a slightly larger ladybug than the native European species. It was originally introduced as a biological combatant against greenhouse pests. It has since spread all over Europe and the US, and it is claimed to potentially have a negative influence on the taste of wine when it invades wine-growing areas. Winegrowers in the US, for example, have reported about wine turning bitter due to a liquid the bugs excrete during stress, that is, for example, while the grapes are being harvested and crushed. The Asian ladybug eats substantially more pests than the native species and reproduces quickly, making it both an effective biological remedy against vine pests and a strong competitor of the local species. 

We drafted four text versions, all of which differed only in how the opening and concluding paragraphs and headlines were phrased, that is, the difference in framing was achieved by variations in only those parts of the text. The opening and concluding paragraphs dealt with the topic of invasive species in general, while the body of the text dealt with the Asian ladybug and was the same across all conditions. The opening paragraph always ended with a sentence stating: “one example of an invasive species is the Asian ladybug”, thereby connecting the differently-framed introduction with the common text body. We used an article originally published in a local viniculture association´s journal as the common body of all texts (number of words: 610) [53]. It dealt with the introduction, characteristics, problems, and possible countermeasures to the Asian ladybug by local vintners. All dependent measures reported in this paper refer to this common part of the text only, unless explicitly stated otherwise.

To establish material close to “real-life” articles, we first searched for articles addressing the topic of invasive species in one local (“Badische Zeitung”) and one national German newspaper (“Die ZEIT”) since 2008. In a second step, we selected articles employing features associated with the human-interest frame. Third, we examined the typical modes of presentation used. Based on these content analyses, we finally defined features for our frames with a dramatizing, emotionalizing, and individualizing emphasis, respectively. Specifically, we defined a frame with dramatizing emphasis as presenting information using dramatizing vocabulary while the general storyline and information remained quite close to the neutral text (i.e., a frame presenting factual information in a tentative way using value-free vocabulary). Our frame with emotionalizing emphasis was based on a representational format used frequently in the newspapers, depicting invasive species almost “xenophobically” as alien intruders, utilizing vocabulary related to negative newspaper coverage on immigration and related topics. Finally, to construct our frame with the individualizing emphasis we used personal quotations and vivid portrayals of the effects of specific invasive species (other than the Asian ladybug). A certain personal relevance was evoked by all four text versions by referring to the region where our participants lived in the headline (“Southern Badenia”; a region in southwest Germany). The introductory and concluding paragraphs followed the same structure in all conditions (employing features as described above): First, we pointed out that animals and plants arriving from other regions are called *invasive species* (*Neobiota* in German). The consequences of invasive species were then described. Finally, all introductory paragraphs ended with a sentence stating: “One/Another example of an (invasive) neobiota is the Asian ladybug (harmonia axyridis).” thereby connecting the differently-framed introduction with the common body of the text. The common body of the text contained four paragraphs titled “Origin and propagation”, “Characteristics”, “Occurrence in vineyards”, and “Potential countermeasures”. The differently-framed concluding paragraphs all stated that there is no way to (completely) stop the immigration of invasive species, naming consequences for the local environment. These consequences once again were depicted according to the respective frame. The lengths of opening and concluding paragraphs were almost identical across conditions, including the neutral-text condition. The average number of words was 130.75 in the opening paragraph (varying between 130 and 131 words) and 59.25 in the concluding paragraph (varying between 56 and 63 words). In addition to the narratives, all texts contained three pictures referring to the text body’s content (one picture of a native ladybug and another of an Asian ladybug, as well as four Asian ladybugs on a plant’s stem). Pictures and the text body were the same across conditions. An English version of the material is obtainable for informational purposes from the corresponding author´s website at http://www.psychologie.uni-freiburg.de/Members/otieno/pub.html (password: plosone). Please note, that the texts, while being carefully translated from the German, do not take into account any cultural specificities, nor did we perform any back-translation as would be necessary to result in a fully comparable English version. 

### Socio-demographic data and the assessment of perception of risk and emotions

The socio-demographic data questionnaire contained items on gender, age, education, and activities in environmental organizations. Perceived risk and emotions were assessed on 5-point scales. Perceived risk of invasive species (termed as “recently immigrated animals and plants”) was assessed before and after reading the text. Emotions and the perceived risk related to the Asian ladybug were assessed immediately after reading the text. 

The questionnaires on perception of risk consisted of seven items based on a questionnaire developed by McDaniels and colleagues [54] and tested by Lazo and colleagues [55]. It included one general item on perceived risk and two items each on McDaniel and colleagues’ identified factors “impact on species” and “impact on humans” as well as one item each on the factors “controllability” and “knowledge of impacts”. A rating of “1” indicated low risk and a rating of “5” indicated high risk for all items. Correlations among all seven items revealed that only five (namely the general risk item and the four items on impact on species and humans) intercorrelated substantially. This finding is in accordance with results of factor analyses by Lazo and colleagues [55]. Therefore, we used a combined measure of the five highly intercorrelated items for our analyses and termed it “perceived risk” (Cronbach’s α = .74 for perceived risk of the Asian ladybug and Cronbach’s α = .81 for the perceived risk of invasive species in general). The items concerning perceived risk of invasive species in general were additionally used to measure perception of risk prior to reading the texts (“prior perceived risk”, Cronbach’s α = .76). 

The questionnaire on emotions consisted of one item addressing general affectedness and four items addressing the specific emotions of anger, sadness, guilt, and fear (1 = *not at all affected/angry/sad/guilty/anxious* to 5 = *very affected/angry/sad/guilty/anxious*). The items, rated immediately after reading the text, were based on those used in the study by Nerb and Spada [56]. The reliability of the combined measure of all items was sufficient (Cronbach´s α = .70). We therefore used the combined measure “negative emotions” for our analyses. 

### Assessment of prior knowledge and learning outcomes

To assess prior knowledge, we asked participants to answer two open questions and six multiple-choice items. The open questions presented prior to reading the text were designed to assess prior knowledge of invasive species in general and the Asian ladybug in particular. Thus, we first asked the participants whether they knew of any invasive species in the region and second, if they had ever heard of the Asian ladybug; if yes, they should tell us what they knew about it. The answers to these open questions were rated on a 5-point scale (0 = *no prior knowledge* to 4 = *very elaborate prior knowledge*). The answers of 32 (i.e., 35.16%) of the participants were rated independently by two raters. Interrater agreement was very good, at ICC = .98 (intraclass correlation coefficient). In the rare cases of divergence, the answers were re-examined together to arrive at a final rating. Given the high interrater agreement, we had just one rater rate the remaining questions. Total prior knowledge was calculated as the sum of points earned in the two open questions (maximum of 8 points for very elaborate answers in both questions) and the six multiple-choice items (maximum of 6 points for all multiple-choice items). 

After participants had read the text, and completed the questionnaires on emotions and perceived risk, we asked them to answer one open question and six multiple-choice items, which were equivalent to the multiple-choice items in the pretest. The open question posed after reading the text was designed to assess the type of information the participants prioritized [57]. We therefore asked the participants the following question: “Which do you consider the most important information about the Asian ladybug - which would you, for example, tell a friend?” To rate the answers to the open questions, we first segmented the answers into units of analysis [58]. These units were assigned to the categories for positive (e.g., “fights pests”), negative (e.g., “invades vineyards”), and neutral (e.g., “is originally from Asia”) statements and for statements referring to information about the Asian ladybug in the text’s body or about invasive species in general in the opening and concluding paragraphs 2 raters coded the answers to the open questions from 32 participants (i.e., 35.16%) independently. Interrater agreement was very good, κ = .87 (Cohen’s Kappa; [59]). In the rare cases of divergence, the answers were re-examined together to arrive at a final consensus. Again, given the high interrater agreement, only one rater rated the remaining questions. Units referring to the text’s opening or concluding paragraph (i.e., differently-framed components) were excluded from further analysis to capture “pure” framing effects (in contrast to content effects). Only units referring to information about the Asian ladybug (i.e., information that was the same across all conditions) were analyzed for prioritization effects. 

Of the six multiple-choice items posed after reading the text, the first asked what invasive species are, that is, it referred to information given in the differently-framed introduction of all four texts. The remaining five multiple-choice items referred to information provided in the body of the text, that is, about the Asian ladybug, which was the same across all conditions. An example of a multiple-choice item about the Asian ladybug is: “What does the Asian ladybug feed on? a) grapes, b) vine pests, c) plant louses, d) grape juice, e) none of the options is correct, or f) I don’t know” (translated from the German). The first item about invasive species served as a control for an appropriate reading of the introductory information. It was not included in the total posttest score on multiple-choice items, resulting in a maximum of five potential points on the multiple-choice posttest. In the pretest, only 7.7% of all participants answered this first item correctly, whereas 98.9% of all participants did so in the posttest. These results reveal that: (1) participants´ prior knowledge about invasive species was very low and (2) the participants had read the opening paragraph carefully, which also enhances the probability of framing effects. 

### Procedure

Data collection took place in a teaching room at the Department of Psychology at the University of Freiburg, Germany. Two trained supervisors assessed groups of up to 20 participants simultaneously. Data on all conditions were collected simultaneously at each session. Materials for each condition were distributed evenly across the seats in the room, that is, each seat was assigned to one specific condition before participants arrived. Participants then selected their own seats when entering the room, which can be considered as a method of random assignment to conditions. Participants were first informed that the study aim was to examine how they perceive a newspaper article concerning invasive species. They were then given an overview of the procedure. They first completed a package consisting of the socio-demographic data questionnaire, prior knowledge test, and the questionnaires on prior risk perception. Participants were then given the respective versions for their seats (i.e., conditions) of the newspaper article, and were asked to read it attentively, as if they had selected it from a newspaper based on vested interest. After reading the text, they were asked to rate their emotions and the perceived risk of invasive species in general and the Asian ladybug in particular. They were then asked to rate the perceived informational value (“How informative do you think the text about the Asian bug was?”; 5-point scale from 1 = *not informative at all* to 5 = *very informative*) and the text's credibility (“How credible do you think the text about the Asian ladybug was?”; 5-point scale from 1 = *not credible at all* to 5 = *very credible*). Additionally, we asked participants which criteria they considered when evaluating the informative value, as well as the credibility: content of the text, presentation of information, or personal opinion on the topic. Finally, participants answered the open question about which information in the text they considered most important and again completed the same multiple-choice test as in the pretest. At the end of each session, participants were debriefed and received their compensation for participation. 

## Results

Framing effects on perceived risk, emotions, and learning outcomes were tested by computing AN(C)OVAs, controlling for *z*-standardized prior perceived risk in the case of perceived risk and for *z*-standardized prior knowledge for quantitative learning outcomes (multiple-choice test; see section “Pre-Analyses”). The assumed mediation effect of perceived risk on negative emotions and the assumed mediation effect of emotions on learning were tested by a set of related multiple regression equations [60], following the bootstrapping approach of Preacher and Hayes [61,62]. We drew 5000 bootstrap resamples as recommended by Hayes [63]. The level of confidence used was 95% and the reported confidence intervals are bias corrected intervals [64].

For all analyses, we used η^2^ as the measure of effect size, with η^2^ of about .01 being considered a small effect, η^2^ of about .06 being considered a medium effect, and η^2^ of about .14 being considered a strong effect [65]. An alpha level of .05 was used for all statistical analyses. 

The authors comply with APA Ethics Code Standard 8.14a, Sharing Research Data for Verification. The data are available to other qualified professionals for confirmation of analyses and results from the authors on request. All raw data will be retained for a minimum of five years after publication.

### Pre-analyses

#### (Prior) perceived risk for invasive species

There was no significant difference in prior perceived risk across the four conditions, *F*(3, 87) = 2.03, *p* = .116. Generally, prior perceived risk was medium in all conditions (*M* = 2.86, *SD* = .63; scale 1-5). Although there were no statistically significant differences, prior perceived risk varied somewhat between conditions on the descriptive level (Table 1). Additionally, there were substantial correlations between prior perceived risk and posttest ratings of perceived risk for the Asian ladybug, *r* = .49, *p*<.001, as well as, not surprisingly, between pre- and posttest measures of perceived risk for invasive species in general, *r* = .55, *p*<.001. To even out potential influences of prior perceived risk of invasive species on perceived risk after reading the information on invasive species and the Asian ladybug, we included the *z*-standardized prior perceived risk measure for all analyses on perceived risk as a control variable. 

**Table 1:** Means and standard deviations of perceived risk, emotions, and learning outcomes.

	Condition/Framing			
		Human-Interest Frame		
	Neutral Text (*N* = 23)	Dramatizing (*N* = 23)	Emotionalizing (*N* = 23)	Personalizing (*N* = 22)
	*M* (SD)	*M* (SD)	*M* (SD)	*M* (SD)
Prior perceived risk, invasive species (1-5)	2.64 (.76)	2.91 (.47)	3.08 (.42)	2.81 (.77)
Prior knowledge (0-16)	.74 (1.29)	1.52 (2.04)	.74 (.96)	.95 (1.25)
Informational value (1-5)	4.17 (.78)	4.26 (.69)	4.22 (.74)	4.05 (.90)
Credibility (1-5)	4.39 (.78)	4.35 (.94)	4.43 (.51)	4.41 (.80)
Perceived risk – post, invasive species (1-5)	2.58 (.79)	3.26 (.79)	3.25 (.77)	3.09 (.59)
Perceived risk – post, Asian ladybug (1-5)	2.28 (.65)	3.23 (.75)	3.38 (.58)	2.99 (.77)
Perceived risk – post, combined (1-5)	2.43 (.66)	3.24 (.72)	3.32 (.55)	3.04 (.65)
Negative emotions (1-5)	1.69 (.67)	2.16 (.73)	2.24 (.61)	2.05 (.62)
General affectedness (1-5)	2.22 (1.17)	2.83 (1.07)	2.87 (1.06)	2.59 (.96)
Learning measures				
Knowledge acquisition- multiple-choice items (0-5)	2.39 (.94)	2.87 (1.01)	3.00 (1.28)	3.00 (.62)
No. of segments (total)	8.91 (5.78)	11.65 (6.42)	10.48 (5.58)	10.18 (3.78)
No. of segments (core text)	8.78 (5.72)	10.26 (6.23)	9.87 (5.36)	9.81 (4.01)
positive statements (%)	11.30 (9.62)	4.06 (6.54)	4.91 (5.72)	4.95 (5.43)
negative statements (%)	32.14 (17.78)	50.23 (20.31)	50.35 (21.40)	37.92 (26.30)
neutral statements (%)	56.56 (16.87)	45.70 (19.25)	44.74 (20.02)	57.13 (26.15)

Perceived risk for invasive species after reading the texts was significantly higher in all human-interest frame conditions compared to the neutral-text condition, *F*(1, 86) = 7.59, *p* = .007; η^2^ = .08. This effect can be seen as a type of manipulation check, as it can be assumed that using dramatizing and emotionalizing vocabulary as well as providing individualized information influences perceived riskiness of a situation. Not surprisingly, we observed no differences between human-interest frame conditions, *F*(3, 64) = .26, *p* = .77 concerning perceived risk of invasive species, which can be considered an initial indicator of our results’ "generalizability" on variants of the human-interest frame. 

#### Prior knowledge

We observed no significant difference in prior knowledge across the four conditions, *F*(3, 87) = 1.51, *p* = .219. Prior knowledge was generally very low (*M* = 0.99, *SD* = 1.46; maximum of 14 points; Table 1). Additional tests for relations between prior knowledge and learning outcomes revealed a significant relation between prior knowledge and posttest scores in the multiple-choice test, *r* = .22, *p* = .038. However, our results revealed no significant relation between prior knowledge and number of segments in the answer to the open posttest question, *r* = .13, *p* = .207, nor in the proportions of positive, *r* = -.03, *p* = 756, negative, *r* = -.02, *p* = .843, or neutral aspects, *r* = .03, *p* = .748 mentioned in answers to the open posttest question. We thus included *z*-standardized prior knowledge as a control variable only in analyses concerning our “amount-of-learning hypothesis”. 

#### Informational value and credibility

To ensure that the different versions of the text did not differ in terms of perceived credibility and informational value, we asked all participants to rate their perceived informational value and credibility on 5-point scales as described under “Procedure”. Analyses of these ratings reveal no difference among conditions with regard to how informative, *F*(3, 87) = .32, *p* = .811, and how credible, *F*(3, 87) = .05, *p* = .984, participants judged their respective version of the text to be. Results suggested that all texts were perceived as quite informative (*M* = 4.18, *SD* = .77; scale 1-5; Table 1) and credible (*M* = 4.40, *SD* = .76; scale 1-5; Table 1) independent of conditions. Additionally, we asked participants for the role various evaluation criteria played when deciding how informative and how credible they judged the text (scale 1-5; 1 = *not at all*, 5 = *very strongly*). More specifically, we asked how strongly their evaluations were based on the content of the text (informative value: *M* = 4.57, *SD* = .58; credibility: *M* = 4.53, *SD* = .66), the presentation of information (informative value: *M* = 3.48, *SD* = 1.08; credibility: *M* = 3.55, *SD* = 1.24), or their personal opinion (informative value: *M* = 2.74, *SD* = 1.26; credibility: *M* = 2.82, *SD* = 1.24). However, there were no significant differences between conditions concerning the evaluation criteria used, nor did the criteria significantly relate to any of the dependent variables, that is perception of risk, emotions, or learning outcomes. 

### Framing effects on perceived risk and emotions

#### Perceived-risk hypothesis

To determine framing effects on perceived risk of the Asian ladybug, we computed ANCOVAs controlling for *z*-standardized prior perceived risk. As expected, a planned contrast showed that the perceived risk for the Asian ladybug was significantly higher in all human-interest frame conditions than in the neutral-text condition, *F*(1, 86) = 25.24, *p*<.001; η^2^ = .23. There were no significant differences among the different human-interest frame conditions with respect to the perceived risk for the Asian ladybug, *F*(3, 64) = .84, *p* = .44 (Table 1).

#### Emotions hypothesis

To determine framing effects on emotions, we computed ANOVAs. As expected, a planned contrast showed that negative emotions referring to the text about the Asian ladybug were significantly stronger in all human-interest frame conditions than in the neutral-text condition, *F*(1, 87) = 8.39, *p* = .005; η^2^ = .09 (Table 1). There was no significant difference among the human-interest frame conditions with respect to negative emotions, *F*(2, 65) = .51, *p* = .60. Additionally, we tested for differences in general affectedness (a potential indicator for arousal): A planned contrast indicated significantly stronger general affectedness in all human-interest frame conditions than in the neutral-text condition, *F*(1, 87) = 4.48, *p* = .037; η^2^ = .05 (Table 1). There was no significant difference among the human-interest frame conditions with respect to general affectedness, *F*(2, 65) = .47, *p* = .63. 

#### Mediation-by-risk hypothesis

**Figure 1:**
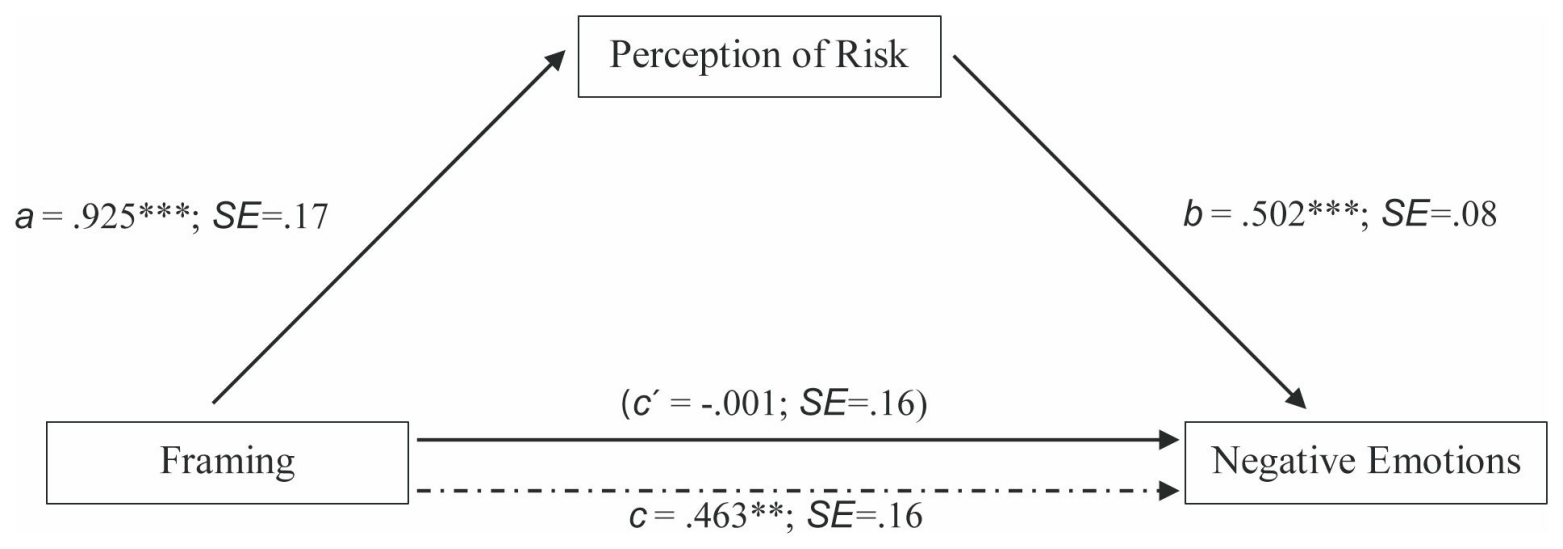
Mediation model: Mediation-by-risk hypothesis. **p =.004, ***p < .001.

Finally, we tested for a mediation effect, that is, whether framing enhanced negative emotions via the increased perceived risk of the Asian ladybug. Results of ANCOVAs of the effect of framing on perceived risk already indicated that framing significantly affected perceived risk. Regression models as calculated in mediation analyses arrived at the same result (path a, Figure 1). The same can be said about the direct effect of framing on negative emotions (path c, Figure 1). Additionally, there was a significant effect of perceived risk on negative emotions controlling for the influence of framing (path b, Figure 1). Finally, bootstrapping analysis for indirect effects indicates that perceived risk can indeed be considered a mediator between framing and negative emotions, a*b = .46, *z*_*ab*_ = 4.06, *p*<.001, η^2^ = 0.10, SE =.11, LCL = .276, HCL = .718. 

Taken together, our results on perceived risk and emotions indicate that all aspects usually found in human-interest frames increased both perceived risk and negative emotions more than a neutral version of the text. Furthermore, our results demonstrate that effects of the human-interest frame can be "generalized" to different variants of the frame, and that the increase in negative emotions was mediated by perceived risk. 

### Framing effects on learning outcomes

#### Amount-of-learning hypothesis

To determine framing effects on learning, we computed ANCOVAs controlling for *z*-standardized prior knowledge. A planned contrast indicated that participants in the human-interest frame conditions performed significantly better on the multiple-choice test than those in the neutral-text condition, *F*(1, 86) = 4.74, *p* = .032; η^2^ = .05 (Table 1). There was no significant difference among the human-interest frame conditions with respect to performance on the multiple-choice test, *F*(3, 64) = .45, *p* = .638. 

#### Prioritization hypothesis

In addition to these quantitative differences in performance on the multiple-choice test, we were interested in analyzing the type of information participants prioritized in their answers to the open questions. Before testing the specific prioritization hypothesis, we conducted ANOVAs comparing the total number of statements in answers to the open question and the number of statements referring to the core text about the Asian ladybug (equal part of the text in all conditions). Planned contrasts revealed no significant differences between the neutral-text and human-interest frame conditions with respect to the total number of statements in their answers, *F*(1, 87) = 1.96, *p* = .165, or the number of statements referring to the core text, *F*(1, 87) = .85, *p* = .36 (Table 1). There were also no significant differences among the human-interest frame conditions with respect to the total number of statements, *F*(2, 65) = .47, *p* = .626, or the number of statements referring to the core text, *F*(2, 65) = .05, *p* = .954.

To test the prioritization hypothesis, we compared the proportions of positive, negative, and neutral statements referring to the core text in answers to the open question. Proportions were calculated as percentages of the number of positive, negative, and neutral statements in relation to the overall number of statements referring to the core text. We only included statements referring to the core text to avoid influences of the differences in the number of aspects covered from the opening and concluding paragraphs between conditions, which may have been due to variations in content rather than pure framing effects. We report proportions as this approach controls for differences in the lengths of answers. The pattern of results was the same for absolute frequencies. 

As expected, planned contrasts showed that participants in the human-interest frame conditions made significantly fewer positive, *F*(1, 87) = 15.35, *p*<.001; η^2^ = .15, and significantly more negative statements, *F*(1, 87) = 7.24, *p* = .009; η^2^ = .08, in their answers to the open question than participants in the neutral-text condition (Table 1). There were no significant differences between the neutral-text and human-interest frame conditions concerning neutral statements, *F*(1, 87) = 2.16, *p* = .145, nor any significant differences among the human-interest frame conditions with respect to positive, *F*(2, 65) = .16, *p* = .850, negative, *F*(2, 65) = 2.20, *p* = .119, or neutral statements, *F*(2, 65) = 2.20, *p* = .119. 

Taken together, our results demonstrate that participants in all human-interest frame conditions prioritized negative over positive aspects when asked to freely recall the most important aspects of the information about the Asian ladybug; positive aspects were largely ignored (c.f. Table 1). This is particularly impressive considering that the information participants read about the Asian ladybug was the same across all conditions. 

#### Mediation-by-emotion hypotheses

Finally, we tested for a mediation effect of negative emotions on learning outcomes. According to MacKinnon [60], a significant relation between negative emotions and learning outcomes controlling for the condition must be identified to establish such a mediation effect. However, we observed no significant relation between negative emotions and learning outcomes when controlling for framing (path b in the mediation model): neither for the multiple-choice test, *B* = -0.02, *SE* = 0.16, *p* = .905, nor for the proportions of negative, *B* = -0.39, *SE* = 3.56, *p* = .912, positive, *B* = -1.49, *SE* = 1.12, *p* = .185, or neutral, *B* = 1.89, *SE* = 3.42, *p* = .582, aspects covered in answers to the open question. We additionally tested for curvilinear relations between negative emotions and the learning outcome measures. However, we detected no significant curvilinear relations. Hence, the hypothesis that framing effects on learning are mediated by emotions is not confirmed. 

Taken together, negative emotions did not seem to play a major role in influencing participants´ understanding of the topic. Rather, our results reveal that it is the framing of the text itself (specifically neutral text versus human-interest framing), which “directly” influenced learning outcomes. 

## Discussion

One crucial aspect of forming opinions and acquiring knowledge is how people perceive and interpret information presented in the media such as newspapers, magazines, information brochures, and the internet, and what and how much they learn from these sources. Journalistic news frames are widely used in these media and are known to influence people’s opinion formation (e.g., in political campaigns and social movements), responsibility attributions, and emotional responses. However, there are few studies directly addressing the effects of news frames on understanding and learning [26]. Our study’s aim was to investigate framing effects on perceived risk, emotions, and learning, as well as their potential mediations combining theories of framing research with theories in cognitive and educational psychology. More specifically, we investigated for effects of the human-interest frame compared to a neutral text independent of content and tested the "generalizability" of these effects by employing different variants of the human-interest frame by emphasizing either the emotionalizing, dramatizing, or individualizing aspect of the frame.

In line with findings in framing research, we found that depicting information in a human-interest frame compared to a neutral text leads to higher perceived risk (perceived-risk hypothesis) and stronger negative emotions (emotions hypothesis). Extending earlier research, our results indicate that these findings can be "generalized" to different variants of the human-interest frame, that is, we can tentatively conclude from our results that human-interest frame effects are indeed effects of the “sum of its parts” and not merely caused by one particular feature. Additionally, our findings show that framing effects on (negative) emotions were mediated by perceived risk (mediation-by-risk hypothesis). Assuming that perceived risk is one aspect of an event's interpretation, this mediation effect is in line with appraisal theories of emotion. Framing effects on perceived risk and emotions in our study might have been influenced by the negative valence of our human-interest frames [66,67]. An additional question to be addressed in future studies therefore is, whether introducing human-interest frames with a positive valence would lead to reduced perception of risk, stronger positive and weaker negative emotions. 

The pattern of results concerning our framing effects on learning outcomes can be interpreted from an educational-psychology perspective by referring to the classic construction-integration model [23,29]. Performance on a multiple-choice test can roughly be considered an indication of the quality of learning on the text-base level, that is, the information directly provided in the text. Answers to the open question reflect understanding on the level of the situation model, that is, the mental model the participants built by enriching the information in the text with their prior knowledge. Most importantly, our findings reveal a potential framing-effects dilemma for learning: Human-interest frames improved the amount of learning on the one hand (amount-of-learning hypothesis), but on the other hand, they led to the prioritization of negative aspects and near ignorance of positive informational aspects (prioritization hypothesis). In other words, the human-interest frame led to better learning of information on the text-base level, yet to a rather “distorted” and – in the sense that it is off-balance – poorly elaborated situation model. This finding is in line with Kintsch and Welsch´s assumption that the situation model is strongly influenced by linguistic cues [25].

Results of the amount-of-learning hypothesis contradict results of Valkenburg and colleagues [4] that human-interest frames, specifically when combined with an emotionally arousing content, hinder learning. This result may be due to only moderate arousal in our study compared to rather high arousal specifically after reading the crime story in the Valkenburg study [38]. This moderate arousal may have enhanced performance also in comparison to participants in our neutral-text condition who rated their general affectedness (a potential indicator for arousal) significantly lower than did participants in the human-interest frame conditions. Additionally, significantly stronger perceived risk and negative emotions in the human-interest frame conditions probably lead to a higher, yet not extremely high, activation level than the neutral-text condition, which is associated with better (quantitative) learning outcomes [68]. 

There might be multiple explanations for the effect on the situation model (prioritization hypothesis): First, the prioritization effect found in our study can be attributed to the framing theories´ assumption that frames narrow down the complexity of an issue by providing a schema for interpretation, that is basically, for building a situation model [18,30,31]. This is also in line with results of Valkenburg and colleagues and Price and colleagues [3,4], who both found that thoughts listed after reading differently framed news stories strongly relate to the respective frame, as well as Shah and colleagues (2004) who found framing effects on associative memory. Human-interest frames with a negative valence as used in our study may provide a schema that directs attention towards negative aspects and away from positive aspects, which relates to the seductive emphasis effect [32,33]. The moderate arousal observed in our study may have further reinforced the prioritization of negative aspects in our human-interest frame conditions [38].

Second, specifically for frames inducing negative emotions, this prioritization effect may also be explained by the personality interaction theory [48], which postulates that negative emotions result in a limited spread of activity and an emphasis on dominant words possibly making people more susceptible to framing effects. In other words, frames with negative valence such as the human-interest frame used in our study potentially lead to a rather narrow processing of information and therefore to a rather one-sided, simplified perception of the topic at hand instead of a balanced and complex representation. Especially in the field of informal learning, when knowledge is acquired outside formal curricula and without direct reliance on a teacher [69], this could be highly problematic. However, as is discussed in more detail below, we did not find any mediation effects of negative emotions on learning. Therefore, it seems questionable, if the effects we found were actually exerted via negative emotions. Possible alternative explanations are discussed below.

Finally, the negative valence of the frame likely influenced the way in which participants assumed the issue of invasive species “should be seen”, that is, the frame could have served as some kind of a social cue manipulating the readers´ perception of the topic [70]. Following this interpretation, the prioritization of negative aspects can be seen as a social desirability bias with participants mentioning those aspects as most important that they considered to be in line with the approved social standard. In other words, the framing effect might be some kind of social desirability effect. 

Additionally, given that long-term memory and the integration of new information mainly rely on the situation model, framing may lead to detrimental effects when processing more information about the same topic in the long run [71]. However, experimental evidence of long-term effects of framing is mixed. Some investigators report that framing effects diminish quickly [72]. Others demonstrate potential long-term effects of framing at least under specific conditions. Lecheler and De Vreese [73] showed that moderate prior (political) knowledge was associated with longer-lasting framing effects than low or high levels of prior knowledge. Lecheler and Matthes [74] found more prolonged framing effects of an emotional frame compared to rational frame. Specifically, the latter effect may have occurred in our study as well, but was unfortunately not tested in a delayed posttest. Putting stronger emphasis on long-term framing effects embedded in a cognitive theory of text comprehension would be worthwhile in future studies. 

Interestingly, in spite of the evidence that differences in framing led to differences in negative emotions, we observed no mediation effect of the emotional state on learning outcome in either the amount of information being learned or the prioritization of different aspects (mediation-by-emotion hypotheses). What most likely happened in our study is that the direct framing effect on learning, that is, the emphasis of some aspects over others, was stronger than the potential effect of elicited emotions. Moreover, this non-existing mediation effect casts doubt on interpretations in earlier studies that human-interest frame effects on learning can mainly be attributed to the emotional nature of the frame [4]. Given our results, it seems more likely that other aspects of features in the human-interest frame influence information processing. Given the dramatizing and emotionalizing nature of the frame and the common use of individualized stories makes issue involvement a promising candidate in this respect. Future studies should therefore attempt to assess more potential mediator variables, such as issue involvement, interest, and (social) attitudes as well as the need-for-approval/ social desirability bias to test for their potential as mediators of framing effects.

### Limitations and directions for future research

The main aim of our study was to investigate the effects of the human-interest frame compared to a neutral-text version of the same information. Additionally, we tested the "generalizability" of the findings to common variants of the human-interest frame in the field. Our attempt to provide ecologically valid material as well as other features of our research design and sample, pose some limitations to our study. 

First, we did not make an experimentally sharp distinction between the features used in human-interest frames. The lack of a sharp distinction resulted from our procedure to arrive at the respective frame versions based on real-life examples. While this approach seems desirable to ensure ecological validity, it led to methodological “fuzziness” with respect to the features commonly used in human-interest frames. Therefore, our material cannot distinguish between the framing effects of particular features of the human-interest frame. However, our aim was not to investigate distinct effects of the features used in human-interest frames. Rather, we attempted to investigate whether emphases in one or the other feature lead to significant differences, that is, if effects are attributable to mainly one feature of the frame or actually to the “sum of its parts”. 

Second, one might argue that despite the explicit reference to the Asian ladybug in measures of emotions and perceived risk, participants may not have been able to really separate their impression of the differently framed parts of the texts and the common core text. Also, the perceived risk of invasive species was likely affected by the core text about the Asian ladybug. This might be especially true as the Asian ladybug was portrayed as an example of an invasive species. However, we still argue that compared to most studies in the field of framing, our attempt enabled us to measure relatively “pure” farming effects.

Third, the relatively small and homogenous sample size of ninety-one university students makes generalization to a larger population difficult. There might be, for example, variance restrictions in our variables due to this sample as well as in moderator variables such as intelligence or socioeconomic status influencing our results. Also, a larger and more heterogeneous sample would allow for more in-depth analyses of the constructs measured in our study using factor analyses as well as SEM analyses for a more detailed investigation of relations between perception of risk, emotions, and learning outcomes. Therefore, future studies should include a more representative and larger sample to test for framing effects. 

Finally, it would be worthwhile to employ more elaborate learning measures to better understand framing effects on learning. One possibility would be to use a posttest developed based on item response theory to be able to differentiate better between difficult and easier test items. Despite these limitations, we are confident that our findings provide valuable insights to explain framing effects on information processing and learning combining theories of media research with cognitive and educational psychology. 

## Conclusion

Journalistic news frames do not just influence people’s perception of a situation in terms of their emotions and perceived risk, they also affect what and how much people learn from the information presented. Our results highlight a potential dilemma concerning the latter. While human-interest frames (independent of their specific implementation) lead to more learning, they also emphasize negative aspects of the information and thus lead to the (mal-)prioritization of these aspects. Such prioritization, together with the neglect of positive aspects, is especially detrimental when learning about topics of conflicting evidence because learners fail to develop a balanced view incorporating different yet potentially valid perspectives. This may be highly problematic in both informal and instructional learning settings because unbalanced views can lead to quite radical positions. Specifically, the introduction of sensational media, such as highly emotional reports and films, in classroom settings should be reconsidered. 

## References

[1] De Vreese CH (2005) News framing: Theory and typology. Information. Des J 13: 51–62. doi:10.1075/idjdd.13.1.06vre.

[2] López WL, Sabucedo JM (2007) Culture of peace and mass media. Eur Psychol 12: 147–155. doi:10.1027/1016-9040.12.2.147.

[3] Price V, Tewksbury D, Powers E (1997) Switching trains of thought: The impact of news frames on readers’ cognitive responses. Commun Res 24: 481–506. doi:10.1177/009365097024005002.

[4] Valkenburg PM, Semetko HA, de Vreese CH (1999) The effects of news frames on readers’ thoughts and recall. Commun Res 26: 550–569. doi:10.1177/009365099026005002.

[5] Iyengar S (1991) Is anyone responsible? How television frames political issues. Chicago, IL: University of Chicago Press.

[6] Gross K (2008) Framing persuasive appeals: Episodic and thematic framing, emotional response, and policy opinion. Pol Psychol 29: 169–192. doi:10.1111/j.1467-9221.2008.00622.x.

[7] Pekrun R, Goetz T, Titz W, Perry RP (2002) Academic emotions in students’ self-regulated learning and achievement: A program of qualitative and quantitative research. Educ Psychol 37: 91–105. doi:10.1207/S15326985EP3702_4.

[8] Um ER, Plass JL, Hayward EO, Homer BD (2012) Emotional design in multimedia learning. J Educ Psychol 104: 485–498. doi:10.1037/a0026609.

[9] Pan Z, Kosicki G (1993) Framing analysis: An approach to news discourse. Pol Commun 10: 55–75. doi:10.1080/10584609.1993.9962963.

[10] Shen F (2004) Effects of news frames and schemas on individuals’ issue interpretations and attitudes. Journalism Mass Commun Q 81: 400–416. doi:10.1177/107769900408100211.

[11] Lecheler SK, de Vreese CH, Slothuus R (2009) Issue importance as a moderator of framing effects. Commun Res 36: 400–425. doi:10.1177/0093650209333028.

[12] Petty RE, Cacioppo JT, Schumann D (1983) Central and peripheral routes to advertising effectiveness: The moderating role of involvement. J Consum Res 10: 135. doi:10.1086/208954.

[13] Von Sikorski C, Schierl T (2012) Effects of news frames on recipients’ information processing in disability sports communications. J Media Psych Theor Methods And Applications 24: 113–123. doi:10.1027/1864-1105/a000069.

[14] Chong D, Druckman JN (2007) Framing theory. Annu Rev Pol Sci 10: 103–126. doi:10.1146/annurev.polisci.10.072805.103054.

[15] Igartua J-J, Moral-Toranzo F, Fernández I (2011) Cognitive, attitudinal, and emotional effects of news frame and group cues, on processing news about immigration. J Media Psych Theor Methods And Applications 23: 174–185. doi:10.1027/1864-1105/a000050.

[16] Van Gorp B, Vettehen PH, Beentjes JWJ (2009) Challenging the frame in the news. Media Psychol 21: 161–170. doi:10.1027/1864-1105.21.4.161.

[17] Kahneman D, Tversky A (1984) Choices, values, and frames. Am Psychol 39: 341–350. doi:10.1037/0003-066X.39.4.341.

[18] Scheufele DA, Iyengar S (2012) The state of framing research: A call for new directions. In: Kenski K Jamieson KH The Oxford Handbook of Political Communication. New York, NY: Oxford University Press.

[19] Entman RM (1993) Framing: Toward clarification of a fractured paradigm. J Commun 43: 51–58. doi:10.1111/j.1460-2466.1993.tb01304.x.

[20] Tankard JW (2001) The empirical approach to the study of media framing. In: Gandy OH Grant AE Reese SD Framing public life perspectives on media and our understanding of the social world. Mahwah, NJ: Lawrence Erlbaum Associates pp. 95–106.

[21] Lang A, Bradley SD, Chung Y, Lee S (2003) Where the mind meets the message: Reflections on ten years of measuring psychological responses to media. J Broadcasting Electron Media 47: 650–655. doi:10.1207/s15506878jobem4704_11.

[22] Johnson-Laird PN (1983) Mental models. Cambridge, MA: Harvard University Press.

[23] Kintsch W, van Dijk TA (1978) Toward a model of text comprehension and production. Psychol Rev 85: 363–394. doi:10.1037/0033-295X.85.5.363.

[24] Scheufele B (2004) Framing-effects approach: A theoretical and methodological critique. Communications 29: 401–428. doi:10.1515/comm.2004.29.4.401.

[25] Kintsch W, Welsch DM (1991) The construction-integration model: A framework for studying memory for text. In: Hockley WE Lewandowsky S Relating theory and data: Essays on human memory in honor of Bennet B Murdock. Hillsdale, NJ, England: Lawrence Erlbaum Associates pp. 367–385.

[26] Kinder DR (2007) Curmudgeonly advice. J Commun 57: 155–162. doi:10.1111/j.1460-2466.2006.00335.x.

[27] Shah DV, Kwak N, Schmierbach M, Zubric J (2004) The interplay of news frames on cognitive complexity. Hum Commun Res 30: 102–120. doi:10.1093/hcr/30.1.102.

[28] Berinsky AJ, Kinder DR (2006) Making sense of issues through media frames: Understanding the Kosovo crisis. J Polit 68. doi:10.1111/j.1468-2508.2006.00451.x.

[29] Kintsch W (1998) Comprehension : A paradigm for cognition. Cambridge, UK: Cambridge University Press.

[30] Scheufele DA (1999) Framing as a theory of media effects. J Commun 49: 103. doi:10.1111/j.1460-2466.1999.tb02784.x.

[31] Scheufele DA (2000) Agenda-setting, priming, and framing revisited: Another look at cognitive effects of political communication. Mass Commun Soc 3: 297. doi:10.1207/S15327825MCS0323_07.

[32] Garner R, Gillingham MG, White CS (1989) Effects of “seductive details” on macroprocessing and microprocessing in adults and children. Cogn Instruction 6: 41–57. doi:10.1207/s1532690xci0601_2.

[33] Otieno C, Renkl A, Liebler K, Deil U, Ludemann T, Spada H (2013) Informing about climate change and invasive species: How the presentation of information affects perception of risk, emotions, and learning. Environ Educ Res (In press). doi:10.1080/13504622.2013.833589.

[34] De Vreese CH (2012) New avenues for framing research. Am Behav Sci 56: 365–375. doi:10.1177/0002764211426331.

[35] Semetko H, Valkenburg P (2000) Framing European politics: A content analysis of press and television news. J Commun 50: 93–109. doi:10.1111/j.1460-2466.2000.tb02843.x.

[36] Neuman WR, Just MR, Crigler AN (1992) Common knowledge : News and the construction of political meaning. Chicago, IL: University of Chicago Press.

[37] Gross K, D’Ambrosio L (2004) Framing emotional response. Pol Psychol 25: 1–29. doi:10.1111/j.1467-9221.2004.00354.x.

[38] Yegiyan NS, Lang A (2010) Processing central and peripheral detail: How content arousal and emotional tone influence encoding. Media Psychol 13: 77–99. doi:10.1080/15213260903563014.

[39] Frijda NH (2009) Emotion experience and its varieties. Emotion. ReView 1: 264–271. doi:10.1177/1754073909103595.

[40] Lazarus RS (1991) Emotion and adaptation. New York, NY: Oxford University Press.

[41] Ortony A, Clore GL, Collins A (1988) The cognitive structure of emotions. Cambridge, UK: Cambridge University Press.

[42] Ellsworth PC, Scherer KR (2003) Appraisal processes in emotion. In: Davidson RJ Scherer KR Goldsmith HH Handbook of affective sciences. Series in affective science. New York, NY: Oxford University Press pp. 572–595.

[43] Yates JF, Stone ER (1992) The risk construct. In: Yates JF Risk-taking behavior. Wiley series in human performance and cognition. Oxford, UK: John Wiley & Sons pp. 1–25.

[44] Renn O, Burns WJ, Kasperson JX, Kasperson RE, Slovic P (1992) The social amplification of risk: Theoretical foundations and empirical applications. J Soc Issues 48: 137–160. doi:10.1111/j.1540-4560.1992.tb01949.x.

[45] Böhm G, Brun W (2008) Introduction to the special issue: Intuition and affect in risk perception and decision making. Judgment Decision Mak 3: 1–4.

[46] Slovic P, Finucane ML, Peters E, MacGregor DG (2004) Risk as analysis and risk as feelings: Some thoughts about affect, reason, risk, and rationality. Risk Anal 24: 311–322. doi:10.1111/j.0272-4332.2004.00433.x. PubMed: 15078302. 15078302

[47] Böhm G (2003) Emotional reactions to environmental risks: Consequentialist versus ethical evaluation. J Environ Psychol 23: 199–212. doi:10.1016/S0272-4944(02)00114-7.

[48] Bolte A, Goschke T, Kuhl J (2003) Emotion and intuition: Effects of positive and negative mood on implicit judgments of semantic coherence. Psychol Sci 14: 416–421. doi:10.1111/1467-9280.01456. PubMed: 12930470. 12930470

[49] Forgas JP (1995) Mood and judgment: The affect infusion model (AIM). Psychol Bull 117: 39–66. doi:10.1037/0033-2909.117.1.39. PubMed: 7870863. 7870863

[50] Nelson TE, Oxley ZM, Clawson RA (1997) Toward a psychology of framing effects. Pol Behav 19: 221–246. doi:10.1023/A:1024834831093.

[51] Davis MA, Chew MK, Hobbs RJ, Lugo AE, Ewel JJ et al. (2011) Don’t judge species on their origins. Nature 474: 153–154. doi:10.1038/474153a. PubMed: 21654782. 21654782

[52] Hulme PE, Pysek P, Nentwig W, Vilà M (2009) Will threat of biological invasions unite the European Union? Science 324: 40–41. doi:10.1126/science.1171111. PubMed: 19342572. 19342572

[53] Wegner-Kiss G, Breuer M (2008) Der Neuling breitet sich aus. Der Badische Winzer: 26.

[54] McDaniels T, Axelrod LJ, Slovic P (1995) Characterizing perception of ecological risk. Risk Anal 15: 575–588. doi:10.1111/j.1539-6924.1995.tb00754.x. PubMed: 7501876. 7501876

[55] Lazo JK, Kinnell JC, Fisher A (2000) Expert and layperson perceptions of ecosystem risk. Risk Anal 20: 179–194. doi:10.1111/0272-4332.202019. PubMed: 10859779. 10859779

[56] Nerb J, Spada H (2001) Evaluation of environmental problems: A coherence model of cognition and emotion. Cogn Emotion 15: 521–551. doi:10.1080/02699930143000013.

[57] Perkins DN, Simmons R (1988) Patterns of misunderstanding: An integrative model for science, math, and programming. Rev Educ Res 58: 303–326. doi:10.3102/00346543058003303.

[58] Chi MTH (1997) Quantifying qualitative analyses of verbal data: A practical guide. J Learn Sci 6: 271–315. doi:10.1207/s15327809jls0603_1.

[59] Fleiss JL, Levin B, Paik MC, Fleiss J (2003) Statistical methods for rates and proportions. 3rd ed. Hoboken, NJ: John Wiley & Sons Inc.

[60] MacKinnon DP (2008) Introduction to statistical mediation analysis. New York, NY: Taylor & Francis Group/Lawrence Erlbaum Associates.

[61] Preacher KJ, Hayes AF (2008) Asymptotic and resampling strategies for assessing and comparing indirect effects in multiple mediator models. Behav Res Methods 40: 879–891. doi:10.3758/BRM.40.3.879. PubMed: 18697684. 18697684

[62] Hayes AF (2013) Introduction to mediation, moderation, and conditional process analysis a regression-based approach. New York, NY: The Guilford Press.

[63] Hayes AF (2009) Beyond Baron and Kenny: Statistical mediation analysis in the new millennium. Commun Monogr 76: 408–420. doi:10.1080/03637750903310360.

[64] DiCiccio TJ, Efron B (1996) Bootstrap confidence intervals. Stat Sci 11: 189–228. doi:10.1214/ss/1032280214.

[65] Cohen J (1988) Statistical power analysis for the behavioral sciences. Hillsdale, NJ: Lawrence Erlbaum Associates.

[66] Claes de Vreese HB (2003) Valenced news frames and public support for the EU. Communications 28: 361–381. doi:10.1515/comm.2003.024.

[67] De Vreese CH, Boomgaarden HG, Semetko HA (2011) (In)direct framing effects: The effects of news media framing on public support for Turkish membership in the European Union. Commun Res 38: 179–205. doi:10.1177/0093650210384934.

[68] Pekrun R (2006) The control-value theory of achievement emotions: Assumptions, corollaries, and implications for educational research and practice. Educ Psychol Rev 18: 315–341. doi:10.1007/s10648-006-9029-9.

[69] Livingstone DW (2006) Informal learning: Conceptual distinctions and preliminary findings. In: Bekerman Z Burbules NC Silberman-Keller D Learning in places: The informal education reader. Counterpoints: Studies in the postmodern theory of education; Vol 249 pp. 1058-1634 (Print); New York, NY: Peter Lang Publishing , Vol. 249. pp. 203–227

[70] Tewksbury D, Jones J, Peske MW, Raymond A, Vig W (2000) The interaction of news and advocate frames: Manipulating audience perceptions of a local public policy issue. Journalism Mass Commun Q 77: 804–829. doi:10.1177/107769900007700406.

[71] Matthes J (2007) Beyond accessibility? Toward an on-line and memory-based model of framing effects. Communications 32: 51–78. doi:10.1515/COMMUN.2007.003.

[72] De Vreese CH (2004) The effects of strategic news on political cynicism, issue evaluations, and policy support: A two-wave experiment. Mass Commun Soc 7: 191–214. doi:10.1207/s15327825mcs0702_4.

[73] Lecheler SK, de Vreese CH (2011) Getting real: The duration of framing effects. J Commun 61: 959–983. doi:10.1111/j.1460-2466.2011.01580.x.

[74] Lecheler SK, Matthes J (2012) Framing effects over time: Comparing affective and cognitive news frames. AZ, USA: Phoenix and Publishing House Available: http://citation.allacademic.com/meta/p_mla_apa_research_citation/5/5/4/4/0/p554401_index.html. Accessed 25 February 2013.

